# Indicators of replicative damage in equine tendon fibroblast monolayers

**DOI:** 10.1186/1746-6148-9-180

**Published:** 2013-09-11

**Authors:** Tina Rich, Livia B Henderson, David L Becker, Hannah Cornell, Janet C Patterson-Kane

**Affiliations:** 1 Institute of Infection, Immunity and Inflammation, College of Medical, Veterinary and Life Sciences, University of Glasgow, 120 University Place, Glasgow G12 8TA, UK; 2 Department of Cell and Developmental Biology, University College London, Gower Street, London WC1E 6BT, UK

**Keywords:** Superficial digital flexor tendon, Tendon, Horses, Cell culture, Polyploidy, DNA damage, DNA repair, Replicative stress, GammaH2AX protein, Comet assay

## Abstract

**Background:**

Superficial digital flexor tendon (SDFT) injuries of horses usually follow cumulative matrix microdamage; it is not known why the reparative abilities of tendon fibroblasts are overwhelmed or subverted. Relevant in vitro studies of this process require fibroblasts not already responding to stresses caused by the cell culture protocols. We investigated indicators of replicative damage in SDFT fibroblast monolayers, effects of this on their reparative ability, and measures that can be taken to reduce it.

**Results:**

We found significant evidence of replicative stress, initially observing consistently large numbers of binucleate (BN) cells. A more variable but prominent feature was the presence of numerous gammaH2AX (γH2AX) puncta in nuclei, this being a histone protein that is phosphorylated in response to DNA double-stranded breaks (DSBs). Enrichment for injury detection and cell cycle arrest factors (p53 (ser15) and p21) occurred most frequently in BN cells; however, their numbers did not correlate with DNA damage levels and it is likely that the two processes have different causative mechanisms. Such remarkable levels of injury and binucleation are usually associated with irradiation, or treatment with cytoskeletal-disrupting agents.

Both DSBs and BN cells were greatest in subconfluent (replicating) monolayers. The DNA-damaged cells co-expressed the replication markers TPX2/repp86 and centromere protein F. Once damaged in the early stages of culture establishment, fibroblasts continued to express DNA breaks with each replicative cycle. However, significant levels of cell death were not measured, suggesting that DNA repair was occurring. Comet assays showed that DNA repair was delayed in proportion to levels of genotoxic stress.

**Conclusions:**

Researchers using tendon fibroblast monolayers should assess their “health” using γH2AX labelling. Continued use of early passage cultures expressing initially high levels of γH2AX puncta should be avoided for mechanistic studies and ex-vivo therapeutic applications, as this will not be resolved with further replicative cycling. Low density cell culture should be avoided as it enriches for both DNA damage and mitotic defects (polyploidy). As monolayers differing only slightly in baseline DNA damage levels showed markedly variable responses to a further injury, studies of effects of various stressors on tendon cells must be very carefully controlled.

## Background

Superficial digital flexor tendon (SDFT) injury affects up to 30-33% of Thoroughbreds and event horses in racing or training (i.e. elite athletes) [1-3]. The consequent failure of regeneration with persistence of scar tissue leads to high rates of both re-injury (up to 50%) and retirement (up to 70%) [4-7]. It is generally agreed that injury usually follows a period of cumulative subclinical microdamage to the extracellular matrix that is both exercise- and age-associated; this damage includes reductions in collagen fibril diameter, increased amounts of weaker type III collagen (type I collagen predominates in normal adult tendon tissue), and reduced glycosaminoglycan levels [8-11].

Tenocytes are tendon fibroblasts with key responsibility for synthesis and degradation of the collagenous and noncollagenous matrix, and are thought to constantly repair microdamage under normal circumstances [12]. In the adult SDFT the tenocytes are not highly active in collagen synthesis or turnover (relative to non-injury-prone digital tendons) [13-15]. This may explain why their reparative capacity is overwhelmed by factors occurring during high-speed exercise, including various potential combinations of high levels of mechanical strain, hypoxia, ischemia-reperfusion events, and hyperthermia (due to hysteresis in energy-storing tendons) [16-20]. Additionally, direct or indirect damage to tenocytes themselves and/or their detachment from collagen fibrils (resulting in mechanical understimulation) can instigate cell death or dysfunction; this dysfunctional activity may include upregulation of matrix metalloproteinase (MMP) activity, increased type III collagen synthesis, and cytokine production [18,21-23]. The result is a vicious cycle of cellular damage and production of mechanically weak matrix that is easily disrupted during normal activity [24].

Due to the limitations of *in vivo* work, appropriate cell culture models are required to more clearly define how tenocytes sense and respond to multiple environmental conditions occurring during galloping exercise, and how these processes might be modulated to reduce injury [25]. Tendon fibroblast monolayer (2-dimensional) culture systems are frequently used as tractable and easily analysed primary systems for experimentation / manipulation [13,21,26]. However, they are also necessary to obtain and expand these cells for use in (currently highly variable and poorly defined) 3-dimensional models, or for clinical purposes e.g. autologous tenocyte implantation into tendon injury sites [26-28]. There are many problems that might influence cellular stress and damage in these monolayers including the tissue extraction process: many researchers use enzymatic digestion rather than explant outgrowth due to the higher and more rapid yield of cells, without significant relative disadvantages in terms of phenotypic drift [13,26-29]. Importantly, levels of such damage can easily go unrecognized when using live/dead assays or simple phase contrast appearance for monitoring, as is common practice [25]. In our monolayers we noted high numbers of binucleate (BN) fibroblasts, a normally rare event in cell culture (excluding cardiomyocytes), that indicates cleavage failure during mitosis and has been associated with DNA damage and matrix surface type [30,31]. This prompted the present study, the objectives of which were to determine: 

(i) a reliable read-out for DNA damage in equine cells;

(ii) the relationship between DNA damage and the replicative fraction;

(iii) whether the relationship between DNA damage and cellular replication altered when fibronectin was used as a surface rather than collagen;

(iv) if reparative activity could overcome any or all of the damage.

Our ultimate aim was to achieve “healthy” tendon fibroblast monolayers i.e. a baseline comprised of cells that were not already responding to stresses introduced by the culture system itself.

## Results and discussion

### Equine SDFT fibroblast monolayers contain abnormally high percentages of binucleate cells, indicating cleavage failure during mitosis

**Figure 1:**
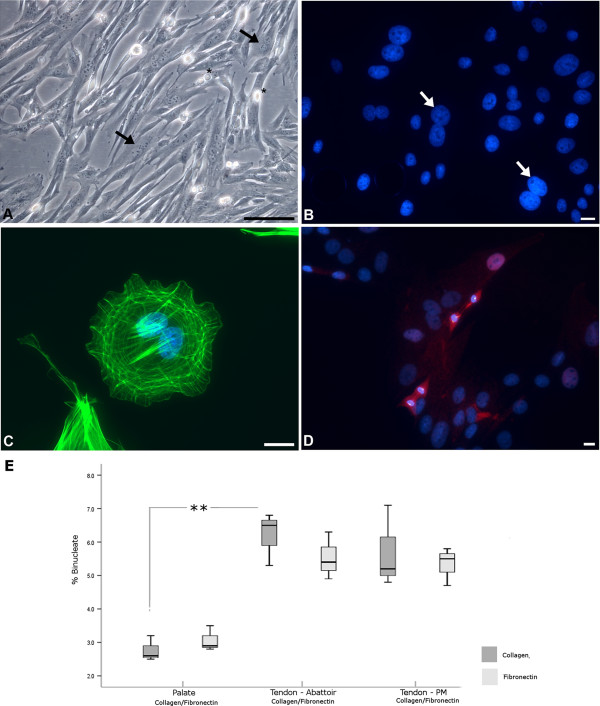
**Binucleate cells in equine tendon fibroblast monolayers.** Phase contrast **(A)** and fluorescent **(B**-**D)** images of primary equine superficial digital flexor tendon (SDFT) fibroblasts, grown on collagen-coated surfaces. **(A)** Numerous binucleate cells in a typical monolayer. The arrows indicate two of the many bi- and multinucleate cells in the field. The asterisks indicate phase-bright mitotic figures just above the focal plane; these are not dead cells. Scale bar = 100 μm **(B)** Nuclei stained blue by DAPI; binucleate cells can most conclusively be identified when the nuclei are touching (arrows). Conservation in size of BN nuclei was commonly seen. Scale bar = 10 μm **(C)** Staining of actin using phalloidin reveals the cytoplasmic boundaries of this binucleate fibroblast. Scale bar = 10 μm **(D)** Two post metaphase dividing cells; this was a common observation and would be unlikely in normal circumstances given that the later phases of cell cycle are shorter than the proceeding prophase and metaphase. A slight dent between the daughter nuclei shows that the cleavage furrow is just beginning to compact the spindle in the lower of the two mitotic figures. The upper cells are further progressed through mitosis, with significant ingress of the central spindle which has compacted the midzone microtubules to form the typical midbody. Cells are labelled with the microtubule-binding protein TPX2 (red; a proliferation marker) and DAPI (blue; nuclear stain). Scale bar = 10 μm. **(E)** Percent binucleation in fibroblasts derived from equine palate or tendon (abattoir and PM group), cultured on either collagen or fibronectin. Cultures were 80% confluent.

Specimens were obtained from an approved UK abattoir (abattoir group), and a veterinary post-mortem facility (post-mortem group; PM). Routine light microscopy examination of culture dishes and phase contrast microscopy of cells seeded onto collagen-coated coverslips revealed large numbers of BN (or occasionally multinucleate) fibroblasts in all monolayers (Figure 1A). In DAPI-labelled monolayers, these could only conclusively be identified where the nuclei were touching unless cytoplasmic or membrane elements were co-stained (Figures 1B,C). In confluent monolayers these comprised up to 7% of the total population and were not related to the age of the animal or the source group (abattoir versus PM) (Table 1). However, in subconfluent cultures the numbers were significantly higher i.e. up to 20%. In all equine tendon fibroblast monolayers, numbers of BN cells were higher than routinely seen in human fibroblast monolayers (~2%) or in equine palate fibroblasts (i.e. primary equine fibroblasts not of tendon-origin) [32], in which they did not exceed 3% (Table 1).

**Table 1:** High binucleate cell numbers and prolongation of late stages of mitosis in tendon fibroblast monolayers

**Source**	**Horse age (y)**	**Binucleate (%)**	**P/M (%)**	**A/T (%)**
Abattoir	5	6.8	0.7	2.8
Abattoir	15	4.5	0.4	2.3
Post-mortem	Foal (stillborn)	4.2	0.4	2.7
Post-mortem	3	5.9	1.8	3.1
Equine palate	(stillborn)	2.9	2.6	2.5
Human Hs68	new-born	1.7	2.9	1.8

The percentage of binucleate cells and those in prophase or metaphase (P/M), or anaphase/telophase (A/T) was calculated from counts of 1000 fibroblasts in ~80% confluent fibroblast monolayers. Values for each parameter in tendon-derived monolayers could not be related to horse age or source. Data from fibroblasts derived from non equine (human Hs68) or equine non tendon (palate) tissue sources are included for comparison.

Binucleation indicates that there has been cleavage failure during mitosis; this is normally a rare event in cell culture for mammalian somatic cells with the exception of cardiomyocytes [30], but can be induced by application of anti-cytoskeletal drugs including cytochalasin B [31,33]. During division a cell rounds up, disassembles the cytoskeleton and redistributes cytoskeletal proteins and other molecules into the cleavage furrow that forms by assembly and contraction of an actomyosin ring to partition the cytoplasm [34,35]. The sister cells then re-spread and separate in the mid-body by removal of cytoskeletal structures from the intercellular bridge, constriction of the cell cortex (furrow ingression), and plasma membrane fission (the process of abscission) [34,35]. In cells where defects occur in cytokinesis (separation of the cytoplasm), furrow regression leads to BN cell formation.

In normal diploid fibroblast monolayers, approximately 70% of the mitotic fraction should be in the early mitotic phases (prophase (P) and metaphase (M)) with the remainder in anaphase (A) and telophase (T) when the daughter nuclei are formed and segregate [36]. Unusually, counts of mitotic indices in tendon-derived fibroblasts revealed a marked enrichment of the later mitotic phases; the A/T counts were 2–7 times those for P/M (Table 1; Figure 1D). This was particularly apparent in low-density cell cultures and was consistent with a delay in proceeding through the latter stages of mitosis. Late phase mitotic enrichment was not evident in human fibroblasts or in fibroblasts derived from equine palate. Previously it was shown that cells that could override a prolonged G2 arrest entered mitosis but remained tethered at the midbody at the end of cytokinesis [37]. Two thirds of those tethered cells became binucleate and the remainder either apoptosed (11%), or re-entered mitosis (22%) whilst still bridged to generate similarly bridged daughter nuclei and eventually, multinucleate cells [37]. Phase contrast images of our monolayer cultures revealed occasional multinucleated cells (Figure 1A), together with the binucleate fraction. The individual nuclei in each BN also showed a high correlation in terms of size (Figure 1B) and frequently the nuclei were enlarged, suggesting two tetraploid nuclei. A mechanism to describe the generation of tetraploid BN cells via successive rounds of failed cytokinesis has been described [38]; this together with the cell tethering described in [37] may be relevant to our monolayer cultures.

Normally, following furrow ingression there is a period when the (now) re-spreading and migrating daughter cells are still linked by the cytoplasmic bridge that narrows and then splits as they move apart [39], i.e. mechanical tension is involved [35]. In one study, 3T3 fibroblasts that were strongly adherent to high-concentration (versus low) fibronectin showed extensive stretching of the intercellular bridge and failure of the midbody to disassemble [33]. It was proposed that cells that cannot move apart from one another due to a too-adhesive substrate will be unlikely to undergo traction-mediated cytofission, i.e. this indicates a significant cell culture artefact [33]. In contrast, researchers using rat embryonic fibroblasts relieved cytochalasin D-induced arrest by growing them on fibronectin, possibly via an integrin-linked effect on the cytoskeleton following failed cytokinesis; it has subsequently been suggested that species-specific differences may contribute to susceptibility to BN formation [40,41]. These differences may be due to altered adhesion properties of fibroblasts on fibronectin versus collagen surfaces, and or their susceptibility to cytoskeletal disruption [42]. To investigate the relationship between failed cytokinesis and matrix substrate for our equine cells, we compared numbers of BN fibroblasts on collagen and fibronectin-coated coverslips but did not see significant differences for these or for palate-derived equine fibroblasts (Figure 1E). If matrix adherence properties are a factor contributing to a greater failure of abscission (separation) for equine tendon fibroblasts versus equine palatine or human foreskin fibroblasts, the difference might also be explained by the difference in tissue-origin; tendon cells in tissue are located within very small niches where they interact intimately with surrounding, densely packed collagen fibrils and other collagenous and noncollagenous matrix components [43].

### Binucleation occurs in tendon fibroblast monolayers regardless of the presence of DNA damage

Binucleation was originally proposed to be a “tetraploidy checkpoint” to protect against cell overgrowth and neoplastic transformation, and therefore invariably resulting in permanent p53-dependent G1 arrest [44]. However other researchers using human diploid fibroblasts showed that the p53 expression causing G1 arrest of BN cells was due to DNA damage (in those experiments caused by cell synchronisation treatments), rather than the tetraploidy itself [31]. It was suggested that cells that have failed to undergo cytokinesis are more likely to arrest because they simply have twice as much DNA in which the damage may be present [31]. The activity of a DNA damage checkpoint to prevent exit from mitosis has since been demonstrated [45]. The full DNA damage response (DDR) is suppressed in normal mammalian cells until the stages of telophase/cytokinesis (i.e. the late stages of mitosis, as enriched in our monolayers), by high activity of cyclin-dependent kinase 1 [46].

Because we documented abnormal accumulation of our equine tendon fibroblasts in the latter phases of mitosis, and due to this known link between DNA damage and binucleation, we immunolabelled the monolayers for the phosphorylated form of the H2AX histone protein variant (γH2AX) using the vertebrate specific monoclonal antibody, clone JBW301; this antibody has previously been used to stain the γH2AX fraction involved in chromatin remodelling in equine sex chromosomes [47]. H2AX is phosphorylated by ATM kinase in response to DNA double-strand breaks (DSBs; the most lethal lesion); this event results in the recruitment of downstream repair factors into a microscopically visible focus [48,49]. An additional kinase, ATR, has been shown to co-operate with ATM/γH2AX to protect collapsed replication forks [50]. The growing γH2AX focus is thought to open up the chromatin structure and forms a “platform” to stabilise accumulating damage response and repair factors; there is close correlation between numbers of foci (or puncta, each containing several hundred γH2AX molecules) and numbers of DSBs [49]. Phosphorylation of H2AX has also been shown in response to stress-induced chromatin reorganisation; this can occur without DNA breaks.

**Figure 2:**
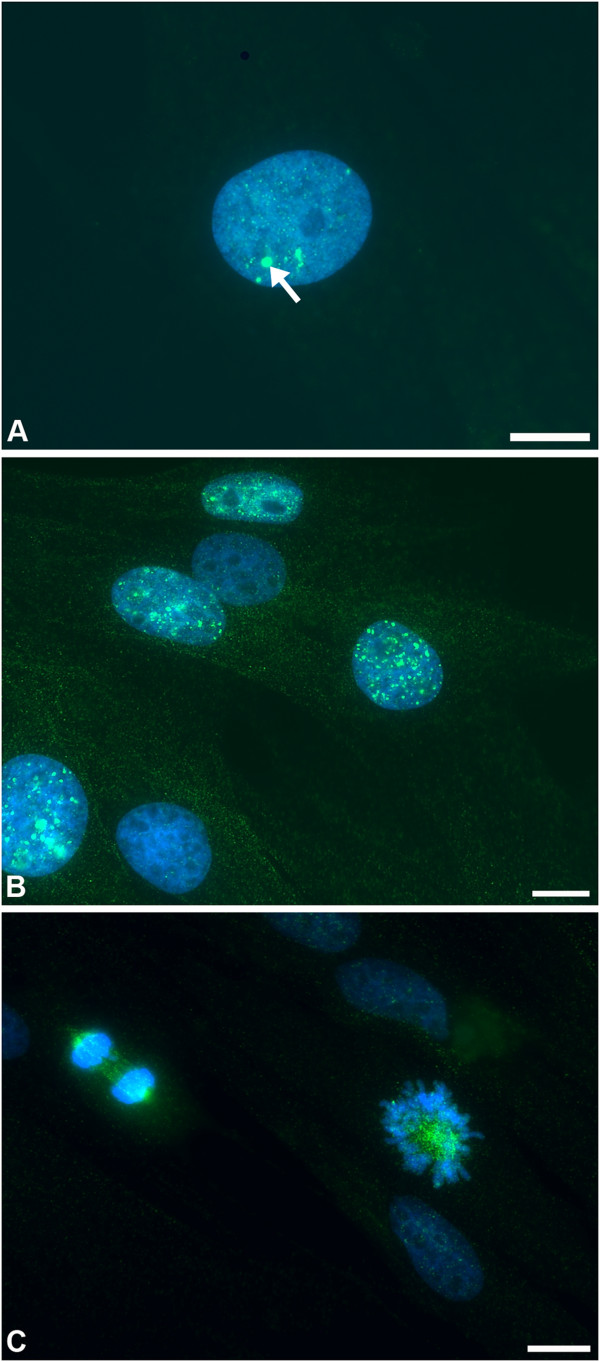
**γH2AX staining patterns in equine tendon fibroblasts. (A)** A fibroblast from the post-mortem (PM) group, in which little damage was noted. A few large γH2AX puncta (green; arrow) are considered an indicator of normal, reparable DNA damage that occurs during the S-phase transition. Numerous micropuncta (green) seen here in the remainder of the nucleus are not thought to be DNA damage-associated. DAPI nuclear counter-stain (blue). Scale bar = 10 μm **(B)** Fibroblasts from the abattoir group, containing remarkable numbers of γH2AX puncta (green) indicating severe DNA damage. Micropuncta can still be seen in the background. DAPI nuclear counter-stain (blue). Scale bar = 10 μm **(C)** Diffuse γH2AX labelling pattern (green) in mitotic fibroblasts, with labelling of the spindle poles and microtubules in the cell on the left. Scale bar = 10 μm.

The S-phase transition in fibroblasts usually results in reparable DNA damage, visible as low numbers of large γH2AX puncta (Figure 2A); we only classified cells expressing 7 or more puncta as excessively damaged. In monolayers from the PM group we detected occasional puncta together with micropuncta of γH2AX (Figure 2A). Micropuncta have previously been shown to arise independently of DNA damage [51], possibly because of the reorganization of chromatin structure that accompanies replication and cell division; only the larger puncta have been associated with DNA damage [49]. In contrast, frequent large puncta were noted in fibroblast monolayers from the abattoir group (Figure 2B), in 8-25% of both BN and mononuclear cells; the highest percentages were seen in the more sparse monolayers i.e. the amount of DNA damage reduced as the fibroblasts became confluent. These levels of γH2AX puncta expression were remarkable, as they have previously only been demonstrated (to this extent) in cells injured using genotoxic agents or ionising radiation [49]. The difference between PM and abattoir groups may have related to the delay of at least 6–7 h prior to placement of specimens into medium (containing digestive enzymes) for the latter group, or subtle differences in digestion technique. We feel that a tissue digestion effect was unlikely, given that comparable levels of DNA damage and binucleation were evident in monolayer and explant cultures derived from the same tissue samples. Percentages of DNA-damaged cells were 15.7% ± 1.6 (mean ± standard error) and 12.8%±2.4 in ~70% confluent monolayer and explant cultures respectively, that were derived from an identical adult abattoir sample. Clearly the speed of cell processing post mortem requires further investigation, particularly as it has been considered acceptable to culture fibroblasts up to several days following death from both human and animal subjects [52,53].

Similar numbers of BN cells were detected in abattoir and PM group monolayers (and explant cultures). This suggested that BN cell formation was largely due to factors other than DNA damage (e.g. matrix substrate, as discussed above). In a previous study of human fibroblasts, not all arrested BN cells contained γH2AX puncta, again suggesting that DNA damage is not a necessary factor [31]. Interestingly, in both abattoir and PM tendon fibroblasts (i.e. those with and without large numbers of γH2AX puncta), diffuse or occasionally punctate γH2AX labelling was noted at mitosis at the spindle poles and microtubules (Figure 2C). In some interphase cells γH2AX labelling was also noted in small perinuclear foci consistent with localisation to the centrosome; this has been reported previously (in tandem with other DNA repair proteins) but is of unknown significance [54]. These distributions of γH2AX (puncta/micropuncta/centrosome-like and spindle) are identical to those previously described in other species [49].

**Figure 3:**
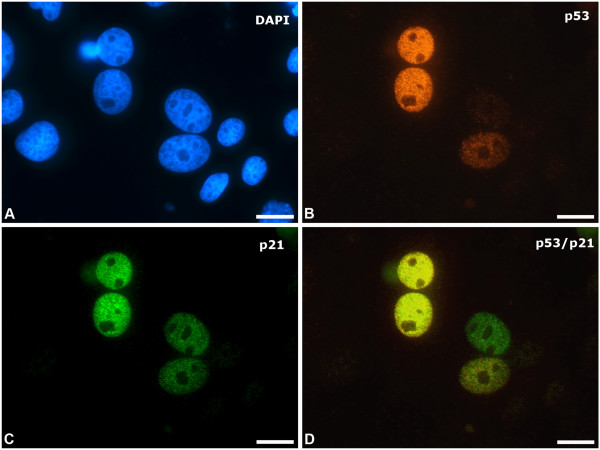
**Cell cycle arrest in binucleate fibroblasts.** These images are taken from the same field **(A)** Two of these fibroblasts are binucleate (BN), with larger nuclei that are almost touching. DAPI nuclear counter-stain (blue) **(B)** Nuclei in one of the BN cells show strong diffuse positive labelling for p53 (red), with faint staining of one of the nuclei in the closely adjacent cell; this indicates stabilisation of p53 to facilitate cell cycle arrest and is not seen in the mononuclear cells **(C)** Both nuclei in both BN cells also show labelling for p21 (green) i.e. the mediator of p53-induced cell cycle arrest **(D)** Colocalisation of p53 and p21. Scale bars = 10 μm.

In abattoir fibroblast monolayers labelled for p53 phosphorylated at serine 15 (a phosphorylation that occurs in response to DNA damage and replication stress), most of the positive nuclei were seen in BN cells i.e. although the numbers of BN formed were not influenced by DNA damage, they were more likely to contain an amount of damage visible in terms of stabilised p53 enrichment (Figure 3A,B). We did not see prominent p53 stabilisation in BN cells in monolayers from the PM group. Additional positive labelling for p21 (cyclin-dependent kinase inhibitor 1) confirmed cell cycle arrest in the p53-positive cells (Figure 3C,D); p21 mediates the cell cycle arrest (at G1), initiated and tightly controlled by stabilised p53. Some researchers have demonstrated the involvement of p21 in elevation of reactive oxygen species (ROS) levels in cultured (human) fibroblasts, feeding back to result in further γH2AX accumulation, continuation of cell cycle arrest and activation of cellular senescence [55]. Not all of the BN cells in the abattoir monolayers had p53-positive nuclei (in some cases only one of the 2 nuclei in one cell contained stabilised p53 (Figure 3B), and some of them were both p53- and p21-negative. Together with the fact that their numbers did not accumulate with time, this indicates survival and eventual re-entry into the cell cycle to complete mitosis. It has previously been demonstrated that if DNA damage does not reach a certain level, most BN cells will eventually finish mitosis and repair the DNA in the next G1 phase [45,46].

### DNA double-strand breaks in tendon fibroblast cultures are replication-associated but do not result in significant cell death

**Figure 4:**
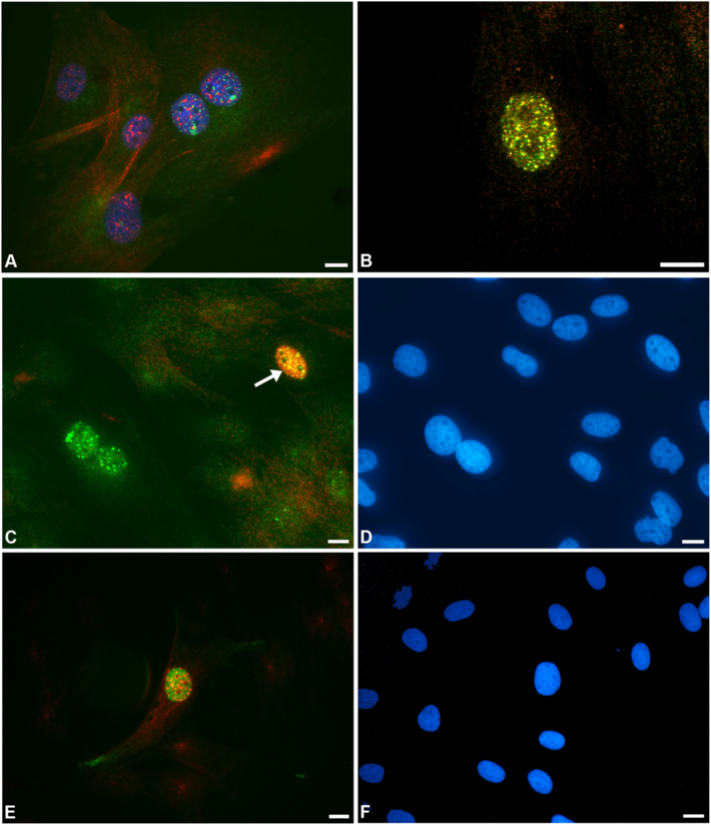
**Association of DNA damage with the replicative fraction. (A)** Promyelocytic leukaemia protein (PML) foci (red) juxtaposed with γH2AX puncta (green); as PML bodies are known to locate next to DNA double strand breaks (DSBs), this confirms that many of the γH2AX puncta in our culture system genuinely indicate the presence of DSBs **(B)** Co-localisation of Rad51 protein (red) and γH2AX (green) as yellow puncta, as further evidence of labelling of DNA breaks. **(C)** Co-localisation (yellow) of γH2AX puncta with positive labelling for the proliferation marker TPX2 in one mononuclear fibroblast (arrow), indicating a correlation between DNA damage and the replicative fraction. A binucleate cell in the same field contains typical, significant numbers of γH2AX puncta but is arrested **(D)** The associated DAPI counter-stain (blue) showing all fibroblast nuclei in the field; note the touching, enlarged nuclei in the BN cell **(E)** The nucleus of a cell that has entered the S/G_2_ phase of the cell cycle, showing diffuse positive labelling for centromere protein F (green); the same nucleus is the only one in this field that contains γH2AX puncta (in this case, red) i.e. significant DNA damage **(F)** The associated DAPI counter-stain (blue) showing all of the fibroblast nuclei. Scale bars = 10 μm.

Because in the abattoir group the numbers of large γH2AX puncta per nucleus were greatest in subconfluent monolayers, we proposed that DNA damage could be occurring in response to replication stress. During DNA replication the replication fork forms a Y-shaped structure at which DNA is unwound and copied. Erroneous replication can stall the replication fork complex, and the single-stranded DNA generated at that point activates cell cycle arrest [56]. If the stalling is prolonged, DNA damage will occur, and if the fork collapses, DSBs are generated that cannot be repaired by simply restarting the fork. However, the precise relationship between the formation of γH2AX puncta and DSBs at blocked replication forks is controversial. Some studies have shown that γH2AX puncta form some hours prior to fork breakage, after many hours of cell cycle arrest, i.e. the puncta may not necessarily represent breaks directly, but they do indicate replication stress and possibly the activities of different repair and chromatin modifying complexes [57,58]. Because we needed to determine if the high numbers of γH2AX puncta in abattoir fibroblasts were actually due to DSBs, we double immunolabelled the cells for γH2AX and two markers of replication-induced DNA damage, promyelocytic leukaemia protein (PML) and Rad51. We previously demonstrated that DSBs juxtapose (without co-localising) with nuclear puncta of PML; PML has since been shown to play a prominent role in the homologous recombination (HR) of DNA during DSB repair [59,60]. This repair process is favoured uniquely in the replicative fraction. The Rad51 protein searches for homologous sequence to act as a template for repair, having first bound to single stranded DNA generated by DNA resection at the breakage site [61]. Our co-staining experiments revealed the stereotypic juxtaposition of nuclear domains of PML protein with large γH2AX puncta in the abattoir fibroblast monolayers (Figure 4A) with co-localised foci of Rad51 (Figure 4B). The majority of the large γH2AX puncta in abattoir cells expressed co-localised puncta of γH2AX and Rad51 (evident in 84-90% of the cells expressing large γH2AX puncta). Rarer, large puncta of γH2AX in PM cells also co-localised with Rad51. However, not all γH2AX puncta contained Rad51 protein; these may have represented (as mentioned above) sites of γH2AX accumulation prior to breakage. Alternatively, some researchers have observed continued marking of resealed but poorly repaired DSB sites by γH2AX in order to recruit the more accurate HR repair machinery [62].

**Figure 5:**
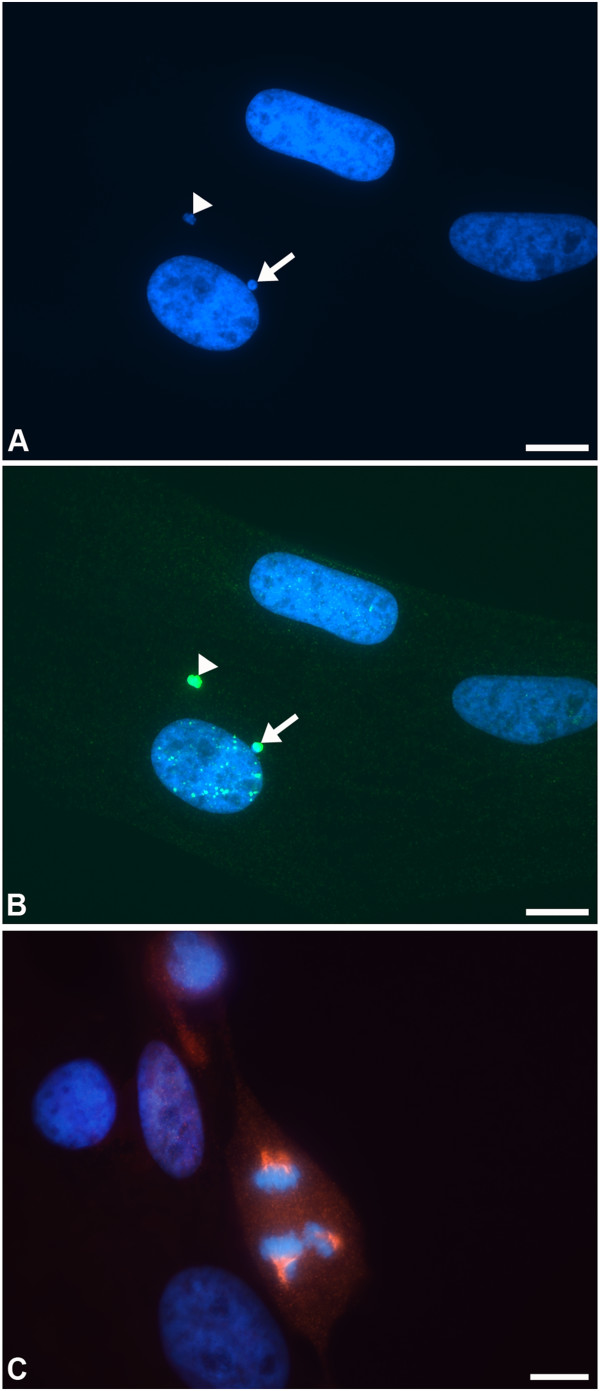
**Morphological evidence of replication stress. (A)** A nuclear bleb or bud (arrow; bleb/buds are nuclear material still attached but derived from the main nucleus) and a microbody (arrowhead; defined as separate or marginally overlapping with the nucleus, less than one third of its size and with a similar DAPI stain (criteria according to [67])); these structures are evidence of DNA damage, and mitotic stress that can give rise to unstable chromosomal material that is ejected from the cell at the subsequent interphase. DAPI counter-stain (blue). **(B)** The nuclear bleb and microbody show positive labelling for γH2AX (green) i.e. DNA damage **(C)** An abnormal, tripolar mitotic figure (TPX2 (red)) with DAPI counter-stain (blue). Scale bars = 10 μm.

**Figure 6:**
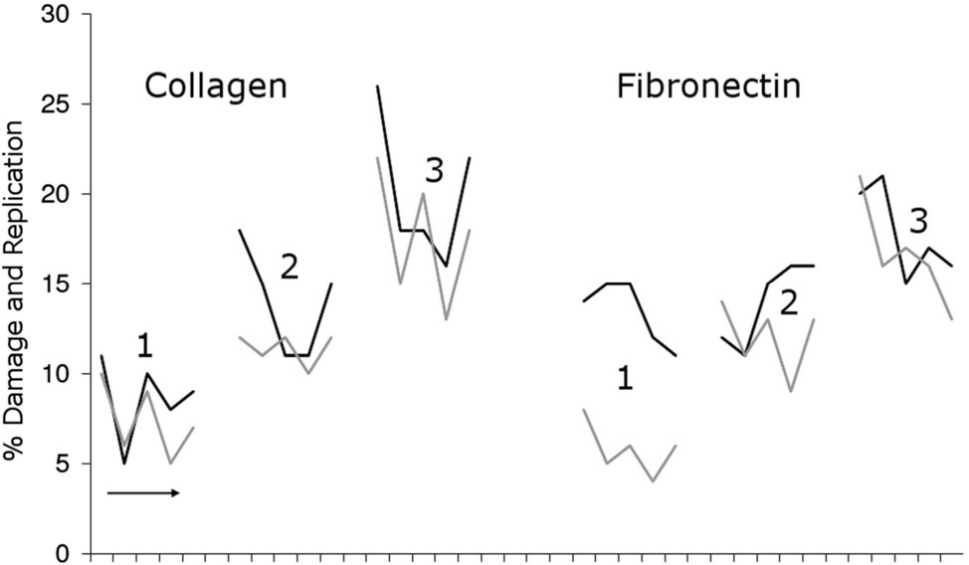
**Close correlation between damaged and replicative fractions in tendon fibroblast monolayers is uncoupled on fibronectin.** The percentages of damaged (black) and replicating (grey) cells are shown in monolayers with low (1), intermediate (2), or high replicative fractions (3) on both collagen and fibronectin surfaces. Each group comprises 5 data points collected from a different source animal. The ages of these animals were, from left to right: 6 months, 3, 5, 14 and 15 years. A close association of the two fractions can be seen on collagen, but on fibronectin the damage level remains relatively high regardless of confluence status i.e. there is an uncoupling of these two events.

Having confirmed a strong association between γH2AX puncta and multiple DNA damage markers in these tendon fibroblasts, we then investigated the link with replication status. Double immunolabelling was performed for γH2AX and TPX2/repp86 or centromere protein F (CENPF) antigens respectively; both of these markers are expressed diffusely in the nucleus during the S and G2 phases of the cell cycle, with relocation to the spindle during mitosis (expression is absent in G1). There was almost exact matching of nuclei expressing increased numbers of γH2AX puncta with positive labelling for each of the replication markers (Figure 4C-F), with the exception of BN cells (arrested in G1). Other evidence of replication stress was noted morphologically, including the generation of nuclear blebs, microbodies, and aberrant multipolar mitoses (Figure 5A-C). There was one exception to this rule, found by comparing replicative and damaged fractions in coverslips from each of the abattoir horses (n = 5) at different levels of confluence on collagen or fibronectin surfaces (Figure 6). An effect of age of the donor (6 months, 3,5,14 and 15 years) was not shown, but for all horses, in the monolayers of the highest confluence level (in which fewer cells were replicating), the damage levels on fibronectin remained high i.e. there was damage-replication uncoupling on that matrix substrate. Confluence was not noted to be reached more rapidly on fibronectin versus collagen. Interestingly, recent data have indicated that fibronectin promotes DNA damage recognition (including H2AX phosphorylation) in an α5 integrin-dependent fashion [63]. This requires further investigation in three-dimensional cell culture systems and in tissue, as it has relevance for cellular therapies: fibronectin expression is markedly upregulated in damaged (and adjacent) tendon tissue, where it occurs pericellularly [64].

Another potential cause of DNA damage detectable by γH2AX staining is *Mycoplasma* sp. infection. Although this damage has never been linked to replicative status, it can be largely reversed by treatment with the free radical quenching agent N-acetyl cysteine (NAC) [65]. We routinely screen our cells for *Mycoplasma* infection and, additionally, we sent cells to a microbiology laboratory for testing (Veterinary Diagnostic Services, University of Glasgow). All isolates were negative. Despite this, we additionally ‘treated’ our cultures with a bacteriocidal agent (plasmocin) that targets mycoplasma protein synthesis and DNA replication, to see whether levels of DNA damage would be reduced. Following several weeks of treatment (i.e. the recommended time to achieve mycoplasma removal) we did not detect a reduction in the levels of γH2AX staining. Treatment with 1 mM NAC also failed to reduce levels of DNA damage. We therefore concluded that DNA damage in our cultures was not because of *Mycoplasma* contamination.

Despite the large amounts of replication-associated DNA damage in the abattoir fibroblast monolayers, there were not similarly significant levels of cell death. Morphologically, few apoptotic bodies were noted, and additionally there was no evidence of the diffuse, nuclear-wide γH2AX labelling that has been associated with apoptosis [57]. Importantly, because most researchers monitor the success of their monolayer cultures by observing and/or measuring cell death and viability, these differences in replication stress and DNA damage would not be identified routinely. We also rarely saw γH2AX puncta in mitotic figures as can occur when collapsed replication forks result in under-replicated loci [66]. These observations indicated that the tendon fibroblasts were largely repairing DNA lesions prior to mitosis.

### Equine tendon fibroblasts have robust DNA repair activity, but the integrity of this repair is uncertain

There were now two reasons for investigating the reparative ability of these tendon fibroblasts: firstly the evidence that DNA damage even at remarkably high levels was not proceeding in most cases to cell death (or permanent arrest of BN cells in those monolayers) i.e. it was being repaired; and secondly due to the potential role of defective repair processes in the formation of DSBs in the first place. Given that SDFT fibroblasts have been shown to have low synthetic and degradative activity levels post-maturation and that they frequently allow accumulation of matrix microdamage [8-15], it was of interest to know if this extended to a poor ability to repair their own DNA.

Because γH2AX labelling only indicates DSB-type damage [49] and repair intermediates we used alkaline comet assays to assess damage and repair in these tendon cells. This assay involves lysing agarose-embedded cells and performing DNA unwinding and electrophoresis at an alkaline pH (12.1-12.4), to detect multiple types of DNA damage i.e. single and double strand breaks, incomplete excision repair sites and cross-links. These lesions often precede DSB formation. DNA migrates out of each cell in response to application of an electric field, and is visualised when fluorescently labelled, as a ‘comet’; the comet tail size and shape correlate with the degree of DNA damage [67]. The equine genome is 2.7 Gb i.e. it is slightly smaller than the human genome at 3.2 Gb. As comet analysis is capable of measuring 0.06 to 3 breaks per 10^9^ daltons, the lowest level of sensitivity would be approximately 100 breaks per equine genome; this is more than adequate to measure levels of background DNA damage in control cells [68]. We used hydrogen peroxide (H_2_O_2_) as a damaging agent because it is known to predominantly cause non-DSB lesions in DNA, particularly in replicating cells. Reactive oxygen species are also of practical relevance in tendon fibroblast research, as they are likely to be increased within the tissue of energy-storing tendons during high-speed exercise [18,19].

**Figure 7:**
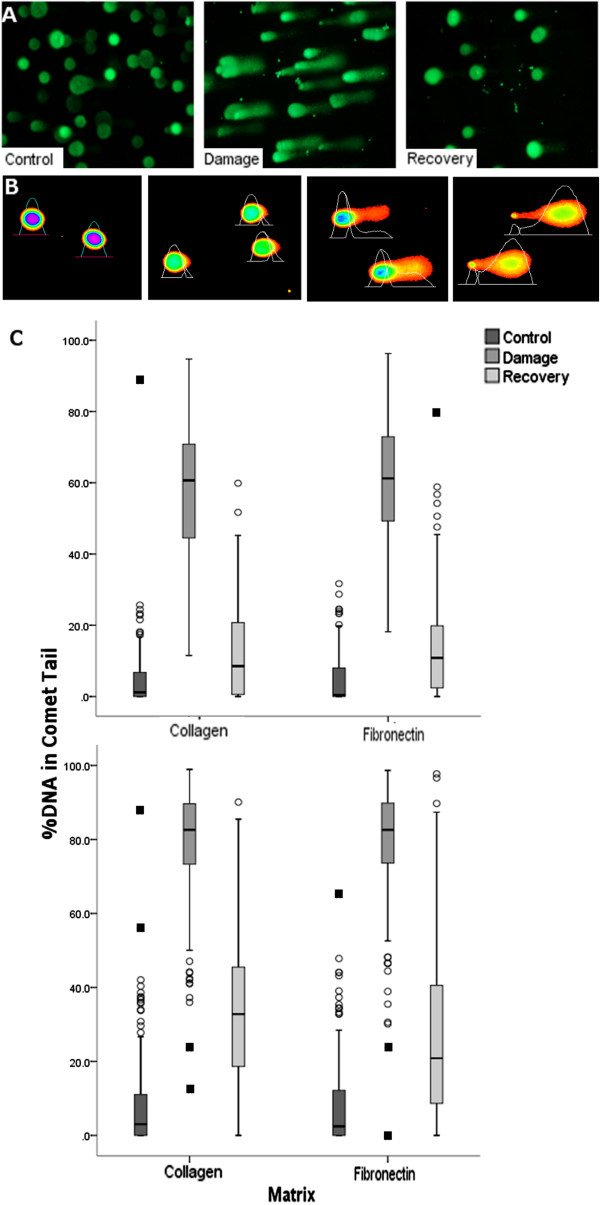
**Comet assays show that rate of DNA repair is linked to initial levels of DNA damage. (A)** Comet assay showing efficient repair of peroxide-induced damage in an abattoir sample. The high level of DNA damage (indicated by long comet tails) in the middle panel is evident in cells collected immediately after peroxide treatment. Much of this damage is repaired after a 1 h recovery period with the result that the comet ‘tails’ are lost in the right hand panel. **(B)** CometScore analyses indicate cells which have been selected from bitmaps; these are converted into histograms by CometScore which quantifies %DNA in comet tails. The cells used in this panel are control cells. These are either undamaged (left hand panel) or have been treated with increasing concentrations of the DNA damaging agent etoposide (left to right). **(C)** CometScore analyses indicate levels of DNA damage following peroxide treatment of two populations from the same horse (PM group). The upper graph shows comet data for EP cells; the lower graph shows comet data for LP cells. The damage induced by peroxide treatment is markedly greater for the LP group; repair of DNA during the recovery period is also delayed for LP cells. Triplicate independent experiments were conducted with at least 50 comets analysed per condition. Outliers at 1.5 box-lengths or more from the edge of the box are indicated by spheres; extreme outliers at 3 or more box-lengths are indicated by black squares. The upper and lower boundaries of the box indicate the interquartile range; the line across the middle of the box represents the median value. Extreme outliers may indicate apoptotic cells or cells that have sustained irreparable damage; wide data distributions indicate these cell fate outcomes together with cells that are repairing, or have repaired, their DNAs.

Significant damage was recorded for both abattoir and PM group cells following H_2_O_2_ treatment, but the majority of comet-tails (indicating broken DNAs) were lost after 1 h recovery i.e. these data showed robust repair activity for both groups (Figure 7A). We were particularly interested to see how subtle variation in initial DNA damage levels could alter damage and repair following the H_2_O_2_ treatment; to achieve this we reduced the biologic noise in our assay by comparing cells derived from the same animal. We used PM cells as they carried a lower amount of endogenous damage. Our assay involved two sets of cells; one early passage (P3) and the second, derived after several weeks in continuous culture (denoted EP/early passage and LP/late passage). To more accurately identify changes in repair capacity we used the CometScore software that allows analysis of individual comets with definition of their DNA distributions (i.e. percentage of DNA in the tail). The CometScore software generates histograms of fluorescence intensity across each comet which can then be quantified (Figure 7B). CometScore analyses from our two groups of cells (early and late passage) are shown in the box-whisker charts in Figure 7C. The upper chart shows EP cells; the lower, LP. The median values for control (initial) levels of DNA damage (% of DNA in the comet tail) in EP and LP cells were 1.2% and 3.0% respectively on collagen-coated surfaces and 0.4% and 2.5% on fibronectin. Following H_2_O_2_ treatment, the median values of these two groups diverged by more than 20% i.e. 60.7% for EP versus 82.6% for LP on collagen, with similar differentials when grown on fibronectin (Figure 7C). This difference was maintained following the 1 h recovery period. We could speculate that the increased injury generated by peroxide treatment of the LP cells delayed repair; the contribution of a minor increase in basal DNA injury prior to peroxide treatment remains to be established. However, if small differences in basal DNA damage can alter the injury thresholds for tendon fibroblasts, researchers will have to be wary of this possibility and carefully control for genotoxic stress in their experiments.

Interestingly, pairwise analyses to compare cells on either matrix surface within each of the EP and LP groups (i.e. comparing the EP controls on collagen and fibronectin) showed that DNA damage levels were not significantly different between cells on collagen versus fibronectin surfaces with one exception; for the LP cells there was a lower DNA damage level of 20.6% in the recovery group on fibronectin, versus 32.8% for collagen (p < 0.001). This finding is in keeping with previous work that indicates potentiation of DNA damage responses in cells growing on fibronectin [63], which might result in the altered repair kinetics that we observed. However, the integrity of this more rapid re-ligation is not certain given our previous measurements of high levels of γH2AX in cells growing on that surface, that occurred regardless of their replication status. High levels of γH2AX labelling have been shown to persist following DNA break re-ligation in situations where DNA repair has been inaccurate [62]. Our demonstration of a DNA damage-replication link would suggest that repair processes that act in replicating cells, for example, HR, may be defective in the tendon fibroblasts. It is possible that this defect is enriched in monolayer cultures of tendon-derived cells due to the fact that they are generally post-replicative in their tissue niche [69]. This possibility will be investigated in future work.

## Conclusions

Culturing equine tendon fibroblasts in monolayers resulted in two forms of cellular injury: numbers of binucleate cells at levels previously only documented in cells irradiated or treated with cytoskeletal poisons; and in cells from some sources, high initial levels of DNA damage including double strand breaks that did not resolve with further replicative cycling but did not result in significant cell death. We demonstrated that both forms of injury were replication-linked on collagen substrates, with uncoupling of the replication-DNA damage link on fibronectin. DNA repair mechanisms were robust, but increased DNA damage delayed repair; this effect was mitigated for cells carrying high levels of DNA damage but cultured on fibronectin. We would recommend that cells expressing these damage phenotypes at an early passage not be injected into injury sites or used for mechanistic injury studies due to problems with comparability. Additionally, ex-vivo cell expansion should be minimised in order to limit replication-induced cellular injury and mitotic disruption. The effects of fibronectin in uncoupling DNA damage from replication and accelerating DNA re-ligation (in monolayers with high DNA damage levels) require further investigation, given the high levels of this matrix glycoprotein in injury sites.

## Methods

### Sample collection

Mid-metacarpal segments (5 cm) of the SDFT were harvested under aseptic conditions from the forelimbs of Thoroughbred horses euthanized for reasons unrelated to tendon disease. Specimens from 5 adults (3, 5, 14, 15 years of age) and one foal (6 months of age) were obtained from an approved UK abattoir. Specimens from one adult (3-year-old) and one foal (full-term stillborn) were obtained from a veterinary post-mortem facility. All specimens were obtained and used in accordance with guidelines reviewed and approved by the Ethics and Welfare Committee, School of Veterinary Medicine, University of Glasgow. Equine palate fibroblasts were donated by Dr. L. Nasir (University of Glasgow) [32]. Human primary foreskin fibroblasts (Hs68) were purchased (CRL 1635; ATCC, Teddington, Middlesex, UK).

### Cell preparation

The paratenon and peripheral tissue were removed from each tendon segment and the remaining tissue diced into 2 mm^3^ pieces. These were incubated with constant agitation for 1 h at 37°C in sterile DMEM medium (10 mL per 1 g tissue) containing 1 mg/mL pronase and supplemented with 10% foetal calf serum (FCS) and 1% penicillin/streptomycin [13,21]. A second digestion for 1 h at 37°C with complete DMEM containing 0.25 mg/mL collagenase type VIII and 0.55 mg/mL dispase (40 mL per 1 g of tissue) was then performed [13,21]. Digested material was passed through a 70 μm cell strainer, and cells collected by centrifugation at 1000 rpm for 5 min; cell pellets were re-suspended in complete DMEM. The collagenase/dispase digestion was repeated to yield more cells. Routine expansion was carried out in flasks coated with 10 μg/cm^2^ type I collagen. For experiments the cells were seeded onto glass coverslips coated with either 10 μg/cm^2^ type I collagen or 1 μg/cm^2^ fibronectin (as per manufacturers’ instructions) and grown to 30-80% confluence in a standard humidified incubator (37°C, 21% O_2_, 5% CO_2_). Equine palate fibroblasts were processed as described previously [32]. Human fibroblasts were seeded onto collagen-coated coverslips in complete DMEM (10% serum; 1 g/L glucose) supplemented with penicillin/streptomycin, and maintained in the same incubator.

### Culture of tendon explants

A 5 cm mid-metacarpal segment of the SDFT were aseptically retrieved and placed into pre-warmed DMEM (37°C). To retrieve tendon fibroblasts from the core of the mid-metacarpal region, a sharp single edged blade (HD Hardware) was used to excise peripheral tissue. The exposed core was diced into 2 mm^3^ pieces that were placed onto collagen-coated 60 mm plastic culture dishes. The bottom of each dish was scored with a scalpel blade to create 5 or 6 parallel lines; this facilitated surface-adhesion of the tissue pieces. Pre-warmed (37°C) complete DMEM containing 10% bovine serum albumin and 1% penicillin / streptomycin was added to the dishes.

### Imaging of binucleate fibroblasts and calculation of mitotic indices

Binucleate (BN) cells were quantified by phase contrast and fluorescent microscopy using an inverted Leica DM IRB microscope; CellMask (plasma membrane dye/Invitrogen) and AlexaFluor 488 Phalloidin were used to demarcate the cytoplasm. Mitotic indices were counted in multiple random fields by fluorescence microscopy using the DNA co-stain DAPI and the replicative marker TPX2. Numbers of prophase (P) and metaphase (M) cells were counted as one cohort and anaphase (A) and telophase (T) as the second.

### Immunocytochemistry

Cells were fixed in 4% paraformaldehyde for 5 min, followed by permeabilization using 0.25% Triton-X 100 for 5 min. Non-specific binding was blocked using 10% milk powder/PBS for 30 min at room temperature (RT). Primary and secondary antibody incubations were carried out overnight at 4°C and 1 h at RT, respectively. Alexa-fluor conjugates (1:1000) were used to detect the primary antibodies. Slides were mounted using Prolong-Gold anti-fade containing DAPI (Invitrogen). Levels of DNA damage were assessed by labelling for phospho-H2AX (γH2AX) (clone JBW301 (1:250) Millipore, Watford, UK). p53 phosphorylated at serine 15 was immunolabelled using ab1431 (1:200; Abcam, Cambridge, UK). Cells were additionally labelled for p21 using EA10 (1:100; Calbiochem, Watford, UK). Double-stranded DNA breaks were more definitively identified by co-labelling for phospho-H2AX and proteins that respond to DNA damage, i.e. either promyelocytic leukaemia protein (PML; H-238 (1:100), Santa Cruz, Dallas, TX, USA) or Rad51 (H-92 (1:100), Santa Cruz, Dallas, TX, USA). Replication was assessed by labelling for the TPX/repp86 and centromere protein F (CENPF) antigens (ab71816 (1:100) and ab5 (1:100), Abcam, Cambridge UK), these being expressed diffusely in the nucleus during S and G2 phases of the cell cycle with relocation to the spindle during mitosis. Cells were examined and imaged using the 63x objective lens of an upright Axiophot2 (Zeiss) or inverted DM IRB microscope (Leica). Fluorescent micrographs were overlaid using GIMP digital software (version 2.6.1).

### Mycoplasma testing

Cell culture samples were added to both solid and liquid mycoplasma media (Mycoplasma Experience, Reigate, UK), and incubated under microaerophilic conditions for up to 7 d. Cell cultures were also tested using the MycoProbe**®** Mycoplasma Detection Kit (R&D Systems Europe Inc., Abingdon, UK). Plasmocin (Invivogen, Toulouse, France) was used at a curative (rather than prophylactic) concentration of 25 μg/mL for two weeks.

### Comet assay

The alkaline comet assay was performed as per the manufacturers’ instructions using triplicate test samples (Trevigen Inc., Gaithersburg, MD, USA). DNA damage was induced by incubation with 200 μM H_2_O_2_ for 5 min on ice. To assess repair capacity, the H_2_O_2_ treatment was followed by a 1 h recovery period. The untreated controls were trypsinized immediately, counted and embedded in agarose. The “treatment” group was processed in this way directly following H_2_O_2_ incubation. The “recovery” group cells were rinsed with PBS, fresh DMEM was added, and they were then maintained for 1 h at 37°C in a 5% CO_2_ incubator prior to processing. The processing was done as rapidly as possible to limit any further DNA repair that would result in under-estimation of the damage at that point. Cells at 1 × 10^5^/mL were added to molten agarose (1:10 v/v). The CometSlides™ were evenly warmed to 37°C prior to application of cells in agarose (to improve adherence of cells); it was important to avoid spreading the cell mixture over the margins of each sample area (delineated in red). The lysis stage was performed at 4°C overnight to increase sensitivity; if the lysis solution crystallized over this period it was discarded. Electrophoresis (21 V, 30 min) at a high pH resulted in structures resembling comets as observed by fluorescence microscopy (using SybrGreen to stain DNA); comets form due to electrophoretic migration of broken DNA strands away from the nucleoid. Electrophoresis is one of the principal factors that can affect data reproducibility. If using a Trevigen CometAssay ES II tank it is crucial to add only 850 mL electrophoresis buffer such that the buffer height is held at 0.4 cm over the slide tray; changing this parameter will alter the efficiency of electrophoresis. Each run was standardized using control ‘damaged’ cells with expected comet tail grades. Images were viewed and captured using a Leica DM IRB microscope. Etoposide-damaged cells (Trevigen Inc., Gaithersburg, MD, USA) were used to establish damage reference groups and ensure that the control conditions were not themselves genotoxic. Treatment and recovery groups were electrophoresed at the same time as controls to reduce experimental variability; failure to visualize comet tails in etoposide-damaged cells was used to indicate failed electrophoresis, in which case the data was not used.

Analysis was performed using CometScore version 1.6.1.11 (TriTek Corp., Sumerduck, VA, USA). Individual “comets” were manually selected from monochrome bitmap images with all metrics saved. Overlapping comets in the images were not scored. Imaging and noise cut-off was as described in [70]. Values plotted were derived from ‘percent DNA in the tail’ as suggested by the COMET assay users group [71]. Each data point represented independent triplicate experiments with at least 50 comets scored per experiment.

### Statistical analysis

All statistical analyses were performed using SPSS Statistics version 9 (IBM, New York, USA). Data normality was assessed using the Kolmogorov-Smirnov statistic. Probability values for significance between the treatment groups were assessed using the non-parametric Mann–Whitney U Test.

## Abbreviations

SDFT: Superficial digital flexor tendon; BN: Binucleate; ATM: Ataxia telangiectasia mutated; ATR: Ataxia telangiectasia and Rad3-related protein; γH2AX: gammaH2AX; MMP: Matrix metalloproteinase; PM: Post mortem; DAPI: 4′,6-diamidino-2-phenylindole; P: Prophase; M: Metaphase; A: Anaphase; T: Telophase; DDR: DNA damage response; DSB: Double-strand break; ROS: Reactive oxygen species; PML: Promyelocytic leukaemia protein; HR: Homologous recombination; CENPF: Centromere protein F; NAC: N-acetyl cysteine; H2O2: Hydrogen peroxide; DMEM: Dulbecco’s modified eagle medium; FCS: Foetal calf serum; RT: Room temperature; LDH: Lactate dehydrogenase.

## Competing interests

The authors declare that they have no competing interests that could inappropriately influence or bias the content of this paper.

## Authors' contributions

TR contributed to study design and creation and acquisition of funds, carried out the cell culture work, immunocytochemistry, microscopic imaging, comet assays, comet analysis, and assisted with statistical analysis. LBH and HC obtained the tendon specimens, derived the cells, and assisted with cell culture, immunocytochemistry, and comet assays. DLB participated in study design, acquisition of funds, data analysis, and drafting and revision of the manuscript. JPK contributed to study design and creation and acquisition of funds, assisted in overall supervision, contributed to statistical analysis, and drafted the manuscript. All authors read and approved the final manuscript.
